# Predictors of unsuppressed viral load among adults on follow up of antiretroviral therapy at selected public and private health facilities of Adama town: unmached case-control study

**DOI:** 10.1186/s12889-022-14169-7

**Published:** 2022-09-19

**Authors:** Fraol Jaleta, Bayissa Bekele, Soriya Kedir, Jemal Hassan, Asnakech Getahun, Tadesse Ligidi, Getinet Garoma, Kiflu Itefa, Tadesse Gerenfes, Abera Botore, Berhanu Kenate, Gutu Dagafa, Daba Muleta

**Affiliations:** 1 Department of Public Health Emergency Preparedness and Research, Adama Public Health Research and Referral Laboratory Center, Adama, Ethiopia; 2 Department of Public Health and Water Analysis, Adama Public Health Research and Referral Laboratory Center, Adama, Ethiopia; 3 Department of Referral Diagnosis, Adama Public Health Research and Referral laboratory Center, Adama, Ethiopia; 4 Department of Capacity Building, Adama Public Health Research and Referral laboratory Center, Adama, Ethiopia; 5 Department of Bio Safety and Bio Security, Ethiopian Public Health Institute, Addis Ababa, Ethiopia; 6 Department of Public Health Emergency Preparedness and Research, Oromia Regional Health Bureau, Addis Ababa, Ethiopia; 7 Department of Anesthesiology, Myungsung Christian Medical Hospital, Addis Ababa, Ethiopia; 8 Chief Executive Officer at Adama Public Health Research and Referral Center, Adama, Ethiopia

**Keywords:** Unsuppressed viral load, Adherence, Case -control, Oromia

## Abstract

**Background:**

Despite the scale up of antiretroviral therapy (ART), unsuppressed viral load among population taking ART in private and public health facilities is still a public health concern increasing the risk of treatment failure. Studies comprehensively assessing significant predictors of non-suppressed viral load among patients on follow up of AR in public and private health facilities are limited. The objective of the study was to identify predictors of unsuppressed viral load among adult patients taking antiretroviral therapy at selected public and private health facilities of Adama town, East shewa zone, Ethiopia.

**Methods:**

An unmatched case-control study was conducted from April 15 /2021 to May 20/2021. A total sample size of 347 patients consisting 116 cases and 231 controls was selected from electronic database among patients who started ART from September 2015 to August 2020. Data were collected using checklist from patient medical records and analyzed by SPSS. The association of dependent and independent variables was determined using multivariate analysis with 95% confidence interval and *P* - value in logistic regression model to identify independent predictors.

**Result:**

From the total 347 participants**,** 140 (40.3%) of them were males and 207 (59.7%) were females. In multivariate logistic regression, CD4 count < 100 [(AOR:1.22, 95% CI: 1.4-7.3)], CD4 100-200[(AOR: 2.58 95% CI: 1.06-8.28)], Fair Adherence [(AOR: 2.44, 95% CI: 1.67-4.82)], poor adherence [(AOR: 1.11, 95% CI: 1.7-6.73)], History of Cotrimoxazole Therapy (CPT) use and not used [(AOR: 2.60, 95% CI: 1.23-5.48)] and History of drug substitution [(AOR:. 361, 95% CI: .145-.897)] were independent predictors of unsuppressed viral load with the *p*-value less than 0.05.

**Conclusion and commendation:**

In this study**,** Baseline CD4, adherence, History of CPT used and history of drug substitution was predictors of unsuppressed viral load. Monitoring immunological response through scheduled CD4 tests is essential to maintain immunity of the patients preventing diseases progression. Intensive adherence support and counseling should conclusively be provided through effective implementation of ART programs by providers would enhance viral suppression ensuring the quality of care and treatment.

## Background

Globally, an estimated 79.3 million people have become infected with Human immunodeficiency virus (HIV) and 36.3 million people have died of Acquired Immunodeficiency Disease Syndrome (AIDS)-related illnesses since start of the disease. In 2020, an estimated 37.7 million people were living with HIV worldwide and 36 million of them were adults. An estimated 68% are living in sub-Saharan Africa [1].

In 2018, almost two thirds (62.1%) of all PLWHIV were receiving life-saving ART, and more than half (53%) had suppressed viral load and nearly half of them had unsuppressed viral load globally. However, the number of people accessing treatment was not rising rapidly enough to reach the 2020 global target of 30 million people. Besides, more than 20% of PLW HIV was not aware of their HIV status [2].

Antiretroviral therapy is aimed to achieve and maintain viral suppression, thereby preventing disease progression and transmission. In 2014, the Joint United Nations Programme on HIV/AIDS (UNAIDS) set the 90-90-90 global targets and for epidemic control of HIV, where by the third 90 represents a target to achieve viral suppression in at least 90% of patients initiating ART by 2020 . The program also set 95-95-95 global target aiming to end epidemic by 2030 in which the third 95 represents a target to achieve viral suppression in at least 95% of patient initiating ART [3].

In 2018, Eastern and Southern Africa accounted 54% of global total HIV infection and 67% had access to antiretroviral therapy (ART) and 46% had unsuppressed viral loads. The study conducted in South Africa also indicates that among 19% of the people admitted to hospital with advanced HIV disease, 21% of admissions were receiving ART with an unsuppressed viral load in 2015 [4, 5].

According to the systematic review of virological efficacy and drug resistance conducted in 2009 sub-Saharan Africa. among 89 studies 15% of patients showed virological failure after two consecutive viral load results of > 1000 copies/ micro litre because of lack of monitoring viral suppression due to inadequate viral load test services [6].

Study conducted in South Africa indicates only 2% of the patients taking first-line ART were switched to second-line ARV despite virology treatment failure ranges from 8 to 17% for patients on ART care in 2012. It was found that there was a delay in assessing, managing, and shifting first line ARV failures [7].

According to the current new spectrum estimate, 665,723 Ethiopians were living with HIV and of which 79.0% of HIV positive adults know their HIV status, 97.1% of them were receiving ART with regional disparities. From Adult positive with HIV receiving ART, 87.6% of them had suppressed viral loads [8].

The number of patients switched to 2nd line ART in Ethiopia remains low which is around 1.5%. This likely reflects the difficulty in determining treatment failure due to limited access of viral load test, and barriers in access to 2nd line regimens [9].

Since 2015, Ethiopian ART guidelines state that Viral load test should be performed for all patients starting from 6 months after ART initiation and then annually for early detection of treatment failure. However, treatment monitoring is still based on clinical and immunological monitoring where there is a limited resource for Viral load test for the decision of treatment failure [9, 10].

Treatment failure among population taking ART in Ethiopia is still a public health concern. According to the study conducted in Ethiopia from March 2016 to 2017, the prevalence of virological failure among population taking ART in Ethiopia is 11% [11].

According to global goal of the three 90s (90-90-90) targets in the development of the current HIV National Strategic Plan, 87% of those on ART have attained viral suppression in Ethiopia [12]. However, viral load testing service coverage which is the gold standard for the decision of treatment failure was 51%.

Systematic review and Meta analysis done in Ethiopia which included 22 published articles from the years of 2012-2018 on magnitude and cause of treatment changes indicates that 7% of the cause of treatment change was treatment failure [13].

Monitoring viral load among individuals receiving ART is important to ensure successful treatment response. Identifying adherence problems and confirmation of ART failure enable clinicians to take an appropriate course of action for patient management [14].

In the absence of viral load monitoring, unnecessary regimen switches are common resulting in increased treatment costs and loss of future options for treatment succession which puts the patient on an increased risk for drug toxicity from second-line regiment [15]. Late detection of treatment failure results in high frequencies of accumulated mutation and drug resistance.

Several studies in public hospitals of Ethiopia indicate that lower CD4, lower Body mass index, Immunological failure, duration in month on ART and adherence associated with unsuppressed viral load. Drug resistance, anti-HIV medications poorly absorbed by the body, Side effect of the medications, other illnesses or conditions are the major impact on treatment success [15–17]. Hence, early detection of non-suppressed viral load is vital for management of the patients and monitoring of treatment outcome.

However, few studies have comprehensively included the patients who follow ART in public and private hospitals as well as in health centers to identify predictors of unsuppressed viral load. Predictors of unsuppressed viral load may vary across different types and levels of health facilities due to the variation in quality of care and treatment. Therefore, this study is aimed to identify factors associated with unsuppressed viral load in both private and public health facilities of the study settings and provide information for implementation of preventive action against factors contributing unsuppressed viral load.

## Methods

### Study design and setting

Facility based cases-control study was conducted at Adama town selected health facilities in East Shewa zone of Oromia among patients enrolled for ART follow up from 2015 to 2020 with data collection period of April 15, 2021 to May 20, 2021. Adama town is located at 8.54^0^N 39. 27°E at an elevation of 1712 m and 99 km away from Addis Ababa with a total population of around 340,000 [18]. There is one government hospital, 8 government health centers, 2 Non-Government health centers and 4 private hospitals with total of 15 health facilities in the town. The study was conducted in five (3 public and 2 private) health facilities namely: Adama hospital medical college, Sr. Aqlishiya Metasabya hospital, Sanfransisco Health center, Gada health center and Adama health center. These selected health facilities started providing ART service in different years. Of them, Adama Hospital Medical College is the first health facility started ART service in 2003. Currently, a total of 2581 adult HIV patients were following first line ART in the selected health facilities and 136 of them had documented viral load of > 1000 copies/ micro liter among first line drug followers [19].

## Study participants

All PLWHIV aged 18 years and above who had been on follow up of ART for at least 6 months were source population. All HIV infected adults who started to ART follow up from 2015 to 20,120 with documented viral load results were study population. The selected cases and controls from the study population were study subjects. All HIV infected adults aged 18 years and above who had history of a single detectable viral load result > 1000 copies/ micro liter at any time after following ART for at least 6 months and above were considered as cases and all HIV infected adults aged 18 years and above who had no history of detectable viral load results > 1000 copies / micro liter were considered as a controls.

### Eligibility criteria

#### Inclusion

- HIV infected patients who were on ART for at least 6 months and above from 2015 to 2020
- Patients who were on First line ART
- HIV infected patients aged greater than 18 years and above

#### Exclusion

- Patients with incomplete data
- Patients who were transfer out

## Sample size determination and sampling procedure

Sample size was calculated using EPi Info version 7.1.1 with 1:2 case and control, 95% CI and power of 80% and using male gender as a key predictor of non-suppressed viral load from previous study [20]. Finally, a total of 347 (116 cases and 231 controls) sample size was calculated for the study. A cases and controls were proportionally allocated to the size of the study population at each health facility. Sample frame of cases and controls was prepared from electronic data base of each hospital using serial and medical record number of the patients. Simple random sampling technique was used to select cases and controls.

## Data collection tools and techniques

Data were collected using checklist developed from ART guidelines of Federal ministry of health of Ethiopia [1, 8] and literatures [20–23] to obtain the necessary data from patients’ records. The checklist containing socio-demographic, medication and clinical related characteristics were designed to review records in to identify the predictors of unsuppressed viral load. From clinical variables adherence level was collected as Good by > 95% (of 30 doses if ≤2 doses were missed), fair by 85-94% (of 30 doses if 3-5 doses were missed) and poor by < 85% (of 30 doses if ≥6 doses were missed) from ART follow up form. Data collectors and supervisor were trained on the content of the tools, objectives of the study, how to extract the data, how to keep and maintain the confidentiality of the patient data and handle the information they obtained. The checklist was pre-tested before the actual data collection was conducted to ensure the quality of the data. Close supervision by supervisor during data collection was carried out and all data were checked for completeness, accuracy and credibility by the principal investigator and supervisors.

## Data analisis

Data were cleaned and entered to EPi Info version 7.1.1 and exported to SPSS version 22.0 for analysis. Univariate analysis was done to describe frequencies, percentages and mean of socio-demographic variables, clinical and drug related characteristics of the study population. Bivariate logistic regression analysis with *p*-value < 0.25 was done to identify candidate variables for significant association of independent variables and outcome variable. All independent variables associated with unsuppressed viral load with *P* < 0.25 were entered to multivariable logistic regression model using Enter method to identify independent predictors of unsuppressed viral load. Independent variables significantly associated with outcome variable with *p*-value < 0.05 in multivariable regression model were considered as independent predictors of unsuppressed viral load. The strength of association between independent variables and dependent variable was determined by Adjusted Odd ratio with a 95% confidence interval. The goodness of the fit for the final model was evaluated using Hosmer -lemshow test and there was no lack of the fit.

## Result

### Socio-demographic characteristics of study participants

From the total of 347 study participants (116 cases and 231 controls), 149 (64.5%) of controls were females. Both males and females were equally accounted 58 (50%) as cases. The median age of study participants was 35 years (IQR 29-42). From the total of cases and controls participated in the study, 84 (72.4%) and 169 (73.2%) of them were orthodox religion followers respectively. 47 (40.5%) of the total cases and 117 (50.6%) of the total controls were married. 98 (42.4%) controls and 49 (42.2%) cases had educational status of primary and secondary school respectively. From the total cases and controls, 100 (86.2%) cases and 193 (83.5%) controls were from urban and 83 (71.6%) of the total cases and 173 (74.9%) of the total controls were unemployed (Table 1).

**Table 1:** Socio demographic characteristics of study participants on follow up of ART at selected public and private health facilities of Adama town, East shewa zone, Oromia, Ethiopia, 2021

Variable	Case (*n* = 116)	Control (*n* = 231)
	Frequency (percentage)	Frequency(percentage)
**Age**		
18-30 years	55 (47.4%)	72 (31.2%)
31-45 years	47 (40.5%)	115(49.8%)
46 -60 years	12 (10.3%)	37 (16%)
> 60 year	2 (1.7%)	(7 3%)
**Gender**		
Male	58 (50%)	82 (35.5%)
Female	58(50%)	149 (64.5%)
**Religion**		
Protestant	14 (12.1%)	30 (13%)
Orthodox	84 (72.4%)	169 (73.2%)
Muslim	18 (15.5%)	30 (13%)
Catholic	–	1 (0.4%)
Other	–	1 (0.4%)
**Marital status**		
Single	37 (31.9%)	38 (16.5%)
Married	47 (40.5%)	117 (50.6%)
Divorced	23 (19.8%)	49 (21.2%)
Widowed	7 (6.03%)	23 (10%)
Separated	2 (1.7%)	4 (1.7%)
**Educational level**		
None (cannot read)	26 (22.4%)	42 (18.2%)
Primary	30 (25.9%)	98 (42.4%)
Secondary	49 (42.2%)	70(30.3%
Tertiary	11 (9.5%)	21 (9.1%)
**Residence**		
Urban	100 (86.2%)	193 (83.5%)
Rural	16 (13.8%)	38 (16.5%)
**Occupational status**		
Unemployed	83 (71.6%)	173 (74.9%)
Employed	33 (28.4%)	58 (25. 1%)
**Substance use**		
Alcohol	1 (0.86%)	1 (0.43%)
Soft and hard drink	2 (1.72%)	–
Chewing khat	2 (1.72%)	4 (1.73)
Never use	111(95.7%)	226 (97.84%)

### Laboratory tests and clinical related characteristics

From the total of case and control participants, about 75 (64.7%) and 153 (66.2%) had baseline hematology parameter respectively and more than half of study participants 253 (73%) had performed baseline CD4 count. Cases and controls with good adherence level accounted 82 (70.7%) and 221 (95.7%) of the total cases and controls respectively. More than half of cases (69.8%) and controls (58.9%) had disclosed their sero-status. 88 (75.9%) and 153 (66. 2%) of case and control had BMI of 18.5-25 kg/m^2^_._ 62 (53.4%) of the cases and 141 (61%) of the total controls had WHO clinical stage I and only 6 (25.6%) and 13 (5.6%) of cases and controls had WHO clinical stage IV. More than half of cases (87.2%) and controls (90%) had history of working functional status (Table 2).

**Table 2:** Laboratory tests and clinical variables of study participants on follow up of ART at selected public and private health facilities of Adama town, East shewa zone, oromia, Ethiopia, 2021

Variables	Case (*n* = 116)	Control (*n* = 231)
	Frequency(percentage)	Frequency (percentage)
**Base line hematology test done**		
Yes	75 (64.7%)	153 (66.2%)
No	41(33.3 5%)	78 (33.8%)
**CD4 measurement done at base line**		
Yes	87 (75%)	166 (71.9%)
No	30 (25%)	65 (29. 1%)
**CD4 result (*N*** **= 253)**		
< 100 cell/micro liter	26 (22.4%)	18 (7.8%)
100-200 cell/micro liter	25 (21.6%)	26 (11.3%)
> 200 cell/micro liter	36 (31%)	122 (52.8%)
**Adherence level**		
Good	82 (70.7%)	221(95.7%)
Fair	13 (11.2%)	2 (0.87%)
Poor	21 (18.1%)	8 (3.5%)
**Disclosure of sero-status**		
Yes	81(69.8%)	136 (58.9%)
No	35 (30. 2%)	95 (41.1%)
**BMI**		
< 18 kg/m2	26 (22.4%)	59 (25.5%)
18.5-25 kg/m2	88 (75.9%)	153 (66.2%)
> 25 kg/m2	2 (1.7%)	19(8.2%)
**Baseline WHO clinical stage**		
Stage I	62 (53.4%)	141 (61%)
Stage II	15 (12.9%)	23 (10%)
Stage III	33 (28.4%)	54 (23.4%)
Stage IV	6 (25.6%)	13 (5.6%)
**Baseline functional status**		
Working	101 (87.2%)	208 (90%)
Ambulatory	12 (10.3%)	20 (8.7%)
Bedridden	3(2.5%)	3 (1.3%)
**History of OI**		
Yes	28 (24.1%)	42(18.2%)
No	88 (73.9%)	189(81.8%)
**History of chronic diarrhea**		
Yes	18 (15.5%)	29 (12.6%)
No	98 (84. 5%)	202 (85.4%)
**History of chronic gastric problem**		
Yes	8 (6.9%)	26 (11.3%)
No	108 (93.1%)	205 (88.7%)

### Health facility and medication related characteristics

From the total of study participants, majority of their address (70%) was a distance of < 10 km away from health facilities**.** Majority of cases (69%) and controls (77.1%) were from government public health facilities. 179 (51%) of the study participants were from the health center and more than half of cases (55.2%) were from hospitals. 79 (68.1%) cases and 191 (82 .7%) controls used TDF + 3TC + EFV regimen at baseline and more than half of cases (82.8%) and controls (79.7%) had used Efavirenz, based first line regimen at base line. 78 (67.2%) cases and 122(52.8%) controls had duration on ART for more than 48 months with the median duration on month 40 (IQR). More than half of case 72.4% and controls (63.2%) had history of drug substitution (Table 3).

**Table 3:** Health facility and Medication related variables of study participants on follow up of ART at selected public and private health facilities of Adama town, East shewa zone, oromia, Ethiopia, 2021

Variable	Case (*n* = 116)	Control (*n* = 231)
	Frequency (percentage)	Frequency (percentage)
**Distance from Health facility**		
< 10 km	73(62.9%)	156(67.5%)
> 10 km	43(37.1%)	75(32.5%)
**Type of health facility**		
Government	80(69%)	178(77.1%)
Private	36(31%)	53(22.9%)
**Level of health facility**		
Hospital	64(55.2%)	104(45%)
Health center	52(44.8%)	127(55%)
**Baseline first line drug regimen used**		
D4T + 3TC + NVP	7 (3%)	1(0.43%)
D4 + 3TC + EFV	8 (6.9%)	1(0.43%)
ZDV + 3TC + NVP	8(6.9%)	1(0.43%)
ZDV + 3TC + EFV	4(3.4%)	22(0.87%)
TDF + 3TC + EFV	79(68.1%)	191(82.7%)
TDF + 3TC + NVP	2(1.7%)	2(0.87%)
TDF + 3TC + DTG	3(2.6%)	3(1.3%)
**Efavirenz based**		
Yes	96(82.8%)	184(79.7%)
No	20(17.2%)	47(20.3%)
Nevirapine **based**		
Yes	15(6.5%)	24(10.4%)
No	101(93.5%)	207(89.6%)
**Adverse effect of drug**		
Yes	5(4.3%)	22(9.5%)
No	111(95.7%)	209(89.5%)
**Duration on ART in month**		
< 24 months	10(8.6%)	36(15.6%)
24ˍ48 months	28(24.8%)	73(31.6%)
> 48 months	78((67.2%)	122(52.8%)
**History of drug substitution**		
Yes	84(72.4%)	146(63.2%)
No	329(27.6%)	85(26.8%)
**History of CPT use**		
Yes	89(76.7%)	135(58.4%)
No	27(23.3%)	96(42.6%)
**History of IPT use**		
Yes	75(64.7%)	171(74%)
No	41(35.3%)	60(26%)
**History of ART drug interruption**		
Yes	33(28.4%)	33(14.3%)
No	83(72.6%)	19(85.7%)

*Abbreviations: D4T* Stavudine, *3TC* Lamivudine, *NVP* Niverapine, *EFV* Efanfirenz, *ZDV* Zidovudine, *TDF* Tenofovir, *DTG* Dolutegravir, *CPT* Co-trimoxazole therapy, *IPT* Isonized Preventive Therapy, *ART* Antiretroviral therapy

### Association of outcome variable and independent variables

In Bivariate analysis, patient characteristics including Gender (COR: 1.82, 95% CI: 1.16-2.86), CD4 count < 100 (COR: 0.24, 95%CI: .101-.414), CD4:100-200 (COR: .307, 95% CI: .158-.596) were significantly associated with unsuppressed viral load. Another clinical and medication related characteristics including fair adherence [COR: 0.057, (95% CI: .013-.258)], poor adherence [COR: .141, (95% CI: .060-.332)], disclosure of sero-status [COR: 0.608,(95% CI: .378-.977)], History of CPT use [COR: 2.34, (95% CI: 1.42-3.88)] and History of treatment interruption [COR: 2.39, (95% CI: 1.38-4.12)] were also candidate for the association with unsuppressed viral load in bivariate analysis (Table 4).

**Table 4:** Bivariate logistic regression analysis of unsuppressed viral load among Adults on follow up of ART at selected public and private health facilities of Adama town, East shewa zone, oromia, Ethiopia, 2021

Variable	Case	Control	COR	95% CI	*P*-value
	Frequency (%)	Frequency (%)			
**Gender**					
Male	58 (50%)	82 (35.5%)	1		
Female	58(50%)	149 (64.5%)	1.82	1.16-2.86	0.01
Age					
18-30 years	55 (47.4%)	72 (31.2%)	1		
31-45 years	47 (40.5%)	115(49.8%)	1.869	1.147-3.045	.012
46 -60 years	12 (10.3%)	37 (16%)	2.355	1.124-4.935	.023
> 60 year	2 (1.7%)	(7 3%)	2.674	.534-13.378	.231
**CD4 result (*N*** **= 253)**					
< 100 cell/micro liter	26 (22.4%)	18 (7.8%)	.204	.101-.414	.000
100-200 cell/micro liter	25 (21.6%)	26 (11.3%)	.307	.158-.596	.000
> 200 cell/micro liter	36 (31%)	122 (52.8%)	1		
**Adherence level**					
Good	82 (70.7%)	221(95.7%)	1		
Fair	13 (11.2%)	2 (0.87%)	1.57	1.32-5.86	0.000
Poor	21 (18.1%)	8 (3.5%)	1.41	1.60-8.32	0.000
**Disclosure of sero-status**					
Yes	81(69.8%)	136 (58.9%)	1		
No	35 (30. 2%)	95 (41.1%)	.608	.378-.977	.040
**BMI**					
< 18 kg/m2	26(22.4%)	59 (25.5%)	1		
18.5-25 kg/m2	88 (75.9%)	153 (66.2%)	.208	.022-1.951	.169
> 25 kg/m2	2 (1.7%)	19(8.2%)	.281	.032-2.440	.250
**History of OI**					
Yes	28 (24.1%)	42(18.2%)	1.432	.834-2.459	.193
No	88 (73.9%)	189(81.8%)	1		
**History of chronic Gastritis problem**					
Yes	8 (6.9%)	26 (11.3%)	0.584	.256-1.334	0.202
No	108 (93.1%)	205 (88.7%)	1		
**Type of health facility**					
Government	80(69%)	178(77.1%)	1		
Private	36(31%)	53(22.9%)	1.51	.918-2.489	.105
**Level of Health facility**					
Hospital	64(55.2%)	104(45%)	1		
Health center	52(44.8%)	127(55%)	.665	.425-1.042	0.075
**Adverse effect of drug**					
Yes	5(4.3%)	22(9.5%)	1		
No	111(95.7%)	209(89.5%)	.428	.158-1.161	.096
**Duration on ART in month**					
< 24 month	10(8.6%)	36(15.6%)	1		
24-48 month	28(24.8%)	73(31.6%)	.724	.724-.317	.443
> 48 month	78((67.2%)	122(52.8%)	.434	.434-.204	.031
**History of drug substitution**					
Yes	84(72.4%)	146(63.2%)	1		
No	329(27.6%)	85(26.8%)	1.528	.939-2.487	.088
**History of CPT use**					
Yes	89(76.7%)	135(58.4%)	1		
No	27(23.3%)	96(42.6%)	2.344	1.416-3.88	.001
**History of IPT used**					
Yes	75(64.7%)	171(74%)	1		
No	41(35.3%)	60(26%)	.642	.397-1.038	.071
**History of treatment interruption**					
Yes	33(28.4%)	33(14.3%)	1		
No	83(72.6%)	19(85.7%)	2.386	1.381-4.120	.002

*Abbreviations: CD4* Cluster Differentiation, *BMI* Body Mass Index, *OI* Opportunistic Infection, *ART* Antiretroviral therapy, *CPT* Co-trimoxazole therapy, *IPT* Isonized Preventive Therapy, *COR* Crude Odd ratio, *CI* Confidence Interval

### Predictors of unsuppressed viral load

All identified candidate variables with *p*-value < 0.25 in bivariate analysis were entered to multivariate logistic regression. In multivariate analysis, CD4 count < 100 [(AOR: 0.122, 95% CI: 0.044-.335)], CD4 100-200[(AOR: 0.258 95% CI: .044-.335)], Fair Adherence [(AOR: .044, 95% CI: .006-.482)], poor adherence [(AOR: .111, 95% CI: .021-.337)],History of CPT used [(AOR: 2.60, 95% CI: 1.23-5.48)] and History of drug substitution [(AOR:. 361, 95% CI: .145-.897)] were independent predictors of unsuppressed viral load with *p*-value < 0.05 (Table 5).

**Table 5:** Multivariate logistic regression analysis of independent predictors of unsuppressed viral load among Adults on follow up of ART at selected public and private health facilities of Adama town, East shewa zone, oromia, Ethiopia, 2021

Variable	Case	Control	COR	AOR	95%CI	*P*-value
	Frequency (%)	Frequency (%)				
**CD4 measurement**						
< 100	26 (22.4%)	18 (7.8%)	2.04	1.22	1.4-7.3	.000**
100-200	25 (21.6%)	26 (11.3%)	3.07	2.58	1.06-.8.28	0.03**
< 200	36 (31%)	122 (52.8%)	1			
**Adherence**						
Good	82 (70.7%)	221(95.7%)		1		
Fair	13 (11.2%)	2 (0.87%)	1.57	2.44	1.67 -4.82	.002**
Poor	21 (18.1%)	8 (3.5%)	1.41	1.11	1.7-6.73	.010**
**History of CPT used**						
Yes	75(64.7%)	171(74%)	1	1		
No	41(35.3%)	60(26%)	2.344	2.60	1.23-5.48	0.012**
**History of drug substitution**						
Yes	84(72.4%)	146(63.2%)	1.53	.361	.014- 089	.028**
No	329(27.6%)	85(26.8%)	1			

*Abbreviations: CD4* Cluster Differentiation, *CPT* Co-trimoxazole therapy, *COR* Crude Odd ratio, *AOR* Adjusted Odd Ratio, *CI* Confidence Interval

**significantly associated at *p*-value < 0.05

## Discussion

Early detection of unsuppressed viral load and identifying its contributing factors prevents treatment failure, drug resistance and minimize transmission of the virus.

In this the study, majority of control 149 (64.5%) were females and both males and females are equally participated as a case 58 (50%).

Several factors were significantly associated with unsuppressed viral load. In multivariate logistic regression, CD4 count, Adherence, History of CPT use and History of drug substitution were the variables independently associated with unsuppressed viral load.

In this study, lower CD4 count was one of the significantly associated variables with unsuppressed viral load. Patients with baseline CD4 < 100 cell /microlitre and CD4 100-200 cell /microlitre were 1.2 and 2.6 times more likely develop unsuppressed viral load than patients with CD4 count > 200 cell /microlitre respectively.

This study is consistent with the study conducted in South Wollo zone of Ethiopia and Northern Ethiopia, Southwestern Ethiopia and Central part of Oromia in which lower CD4 count was significantly associated with viral suppression status [15, 20–22]. This implies that when CD4-count is very low, the immune system no longer strong enough to fight back infections and the viral load continuous to rise.

This study is not similar with the study done in Morocco in which baseline CD4 count has no significant association with unsuppressed viral load and also not similar with the study done in South Africa in which the odd of developing non-suppressed viral load or virological failure was low among patients with higher CD4 count compared to those with low CD4 count [24, 25].

The possible reason might the difference in sample size and, inclusion criteria and study design which was retrospective cohort study with big sample size in this study compared to our study.

Adherence level was also significantly associated with unsuppressed viral load. Patients with fairly and poorly adhered to their medications were 2.44 and 1.11 times more likely to have unsuppressed viral load in their blood than those patient who well adhered to their medications.

This is consistent with the studies conducted in Northern Ethiopian, South west Ethiopia, Oromia and Rwanda in which fair and poor adherence level were significantly associated with non-suppressed viral load compared to patients with good adherence [15, 21, 22, 26, 27].

This indicates that failure of being adherent to daily intake of medication could trigger multiplying of the virus increases the risk of mutation and drug resistance leading to mortality and morbidity of HIV infected patients.

However, this study is not similar with the study conducted in Weliso town of Oromia, Ethiopia, Vietnam and Ghana in which adherence level had no statistically significant association [28–30]. The reason for the variation might be the difference in the method of data collection which included laboratory testing in other area and the difference in the study design compared to other study.

History of CPT use was other predictors of unsuppressed viral load. Patients who had not history of CPT use were 2.60 times more likely to develop unsuppressed viral load than those who had not history CPT.

This study is similar with the study conducted in South Africa and Amhara regional hospitals in which CPT users were one of the independent predictors of virological failure or non suppressed viral among patients on follow up of first line ART treatment [31, 32]. Cotrimoxazole preventive therapy is used to restore immunity lowered due to pneumocystis pneumonia and other infections in HIV patients. If immunity is not maintained, there is the risk of mortality and morbidity due these infections enhancing the replication the virus.

This study is not supported with the study done in Waghimra of Northern and Northeast Ethiopia in which history of CPT use was not significantly associated with virological failure [27, 33]. The possible reason might be due to the sampling technique and the type of study facility which was only based on public hospitals in the above studies compared to our study in which both private and public health facilities were included.

History of drug substitution protectively associated with unsuppressed viral load. The odd of unsuppressed viral load is 63.9% lower among the patients who had not history of drug substitution compared to those patients who had history drug substitution.

This study is supported with study conducted in Western Kenya in which history regimen change/substitution was significantly associated with virological failure and higher among patients who had no history of drug substitution compared to those who had history of drug substitution [34]. The possible reasons for drug substitution or modification are; drug toxicity, drug-drug interaction, comorbidities and treatment failure. Unless regimen changed / substituted due to these possible causes is well monitored, there could be a concern of medication intolerance, adherence, and drug resistance which subsequently affect viral suppression. However this study is not consistent with study done in Bahir Dar in which regimen change has no any significant association [35]. The possible reason might be the difference in inclusion criteria and the type of the health facilities included in the study which might differ in quality of care and treatment. The study conducted in Bahir Dar included only public health facilities compared to this study in which public and private health facilities were comprehensively included in the study.

### Limitation of the study

Since this study was conducted based on record review of the secondary data, the reliability of the data could be affected and selection bias might be occurred because of unmatched selection of case and control. The important variables (socio-economic and psycho-social) not available on the records which could affect the adherence of patients to their medication were not included in the study. Since mutation of the virus (genetically changed) is one of the possible causes of non-suppressed viral load, there was no evidence of drug resistance test due to absence of the testing service. In this study, the cases was selected based on single detectable viral load result of > 1000 copies/micro liter observed at any time after 6 months on ART, we did not evaluate the occurrence of treatment failure.

## Conclusion and recommendations

In this study, decreased CD4 count, poor adherence, no history of CPT use and history of drug substitution were significantly associated with unsuppressed viral load. Prompt diagnosis and early initiation of ART is very essential to monitor immunological response and prevent replication of the virus. Intensive adherence support and counseling should conclusively be provided to the patients by ART clinic team and case team managers of the health facilities. Factors that initiate drug substitution such as drug toxicity, side effect and drug stock out have to be monitored by health care provider to ensure sustainable viral suppression. Effective implementation of ART programs by providers and partners could monitor the quality of care and service provided in the facilities.

## Data Availability

The data of this work are available from corresponding author up on reasonable request with the permission of Adama public health research and referral laboratory center research committee. Corresponding author address for data access: firanoljako1387@gmail.com

## Abbreviations

AIDS: Acquired Immune Deficiency syndrome
APHHRLC: Adama Public Health Research and Referral Laboratory Center
ART: Antiretroviral Therapy
CD4: Cluster Differentiation
EFV: Efanferenzi
FMOH: Federal Ministry of Health
HAART: Highly Active Antiretroviral Therapy
HIV: Human ImmunoVirus
3TC: Lamivudin
NVP: Niverapin
RNA: Ribo nucleic acid
ORHB: Oromia Regional Health Bureau
PLWHIV: People Living with Human Immuno virus
d4T: Stavudin
DGT: Dolutegravir
TDF: Tenofovir
VL: Viral Load
ZDV: Zidovudine
